# Etiology of patients with extreme thrombocytosis and its association with coagulation function: 10-year experience in a tertiary hospital

**DOI:** 10.3389/fmed.2025.1650704

**Published:** 2025-10-23

**Authors:** Huadong Chen, Shuying Huang, Gongqiang Wu, Zhenjiao Zhang, Zhenwei Yu, Qiaoyun Chen, Manzhen Ying

**Affiliations:** ^1^Pharmacy Department, Affiliated Dongyang Hospital of Wenzhou Medical University, Dongyang, China; ^2^Hematology Department, Affiliated Dongyang Hospital of Wenzhou Medical University, Dongyang, China; ^3^Research Center for Clinical Pharmacy, Sir Run Run Shaw Hospital, School of Medicine, Zhejiang University, Hangzhou, China; ^4^Emergency Department, Affiliated Dongyang Hospital of Wenzhou Medical University, Dongyang, China

**Keywords:** extreme thrombocytosis, coagulation function, platelet, etiology, prothrombin time

## Abstract

**Background:**

Identifying the cause of extreme thrombocytosis is important, but few data exist. This study aimed to analyze the etiology of extreme thrombocytosis and its influence on coagulation function.

**Methods:**

We performed a ten-year retrospective study that included patients with platelet counts >1,000 × 10^9^/L. The etiology of extreme thrombocytosis was analyzed by physicians and compared between subgroups, which were stratified by department, age and sex. We also collected the coagulation function of inpatients, including the prothrombin time (PT), international normalized ratio, partial thromboplastin time ratio, prothrombin activity, activated partial thromboplastin time (APTT), fibrinogen, thrombin time and D-dimer, when patients were under extreme thrombocytosis.

**Results:**

Overall, 437 patients were included in the study, including 254 inpatients, 125 outpatients, and 58 emergency patients. The most common cause was myeloproliferative neoplasms (MPN), followed by infection and asplenia. However, the etiology of disease in nearly 10% (41/437) of patients is unclear. MPN was the main etiology for patients from different departments, but the second most common etiologies for inpatients, outpatients and emergency patients were infection, chronic myelogenous leukemia, and unknown, respectively. The most common cause in children is infection. There was no difference between females and males, except for asplenia. Among the inpatients, 87.5 and 60.1% of the patients had PT and D-dimer higher than the normal range, respectively. Moreover, APTT and FIB were in critical condition for 11.1 and 13.7% of the patients, respectively.

**Conclusion:**

This study provides the etiology of extreme thrombocytosis and its association with coagulation function. This study could benefit the future diagnosis and treatment of extreme thrombocytosis.

## Introduction

Thrombocytosis is a frequent, reactive phenomenon caused by a variety of factors (1). The diagnosis of thrombocytosis requires sustained thrombocytosis of >450 × 10^9^/L (2, 3). In terms of etiology, thrombocytosis can be divided into primary thrombocytosis and secondary thrombocytosis. Primary thrombocytosis is a chronic myeloproliferative disease characterized by continuous hyperplasia of bone marrow megakaryocytes and abnormal thrombocytosis. It is a clonal proliferative disease of hematopoietic pluripotent stem cells (4, 5). However, the most common type of thrombocytosis is secondary (reactive) thrombocytosis, which can be due to infection, trauma, surgery, or occult malignancy (3, 6).

In terms of severity, thrombocytosis can be classified by the peripheral platelet count as mild, moderate, severe, or extreme. A platelet count (PLT) ≥ 1,000 × 10^9^/L can be defined as extreme thrombocytosis (3). Approximately 25% of primary thrombocythemia patients present with extreme thrombocytosis (7). Although the cause of thrombocythemia has been well-studied, the data about etiology of extreme thrombocytosis is relatively rare (8). Previous studies have shown that surgical complications and hematological malignancies are the two main causes of extreme thrombocytosis in children and may have features that are distinct from those in adults (9). Extreme thrombocytosis is much less common in children than in adults (10).

Coagulation function is a concern when extreme thrombocytosis develops, as bleeding events or thromboembolism events following extreme thrombocytosis could be life-threatening (11, 12). A combination of coagulation tests (prothrombin time, activated partial thromboplastin time, and fibrinogen) with platelet count and bleeding time would offer the most informative view of a patient's hemostatic process. It has been reported that activated partial thromboplastin time is slightly prolonged, the prothrombin index is slightly shortened and thrombin time is normal, whereas fibrinogen is mildly decreased in essential thrombocythemia patients (13). However, few studies have investigated the effects of extreme thrombocytosis on coagulation function.

Thus, we performed this study to assess the etiology of patients with extreme thrombocytosis in different population subsets during a 10-year period. We also analyzed the coagulation function of extreme thrombocytosis patients to provide information for better management in the future.

## Materials and methods

### Study design and ethics approval

This was a single-center, retrospective study which was carried out in accordance with Declaration of Helsinki. Ethic approval was obtained from the Ethic Committee of Affiliated Dongyang Hospital of Wenzhou Medical University (Reference number 2022-YX-054). The requirement for obtaining informed consent from the patients was waived because of the retrospective nature of the study.

### Patient inclusion and data collection

This study retrospectively included all patients with platelet counts over 1,000 × 10^9^/L who were admitted to Affiliated Dongyang Hospital of Wenzhou Medical University between January 2011 and December 2021. We collected patients' demographic data and individual laboratory test results from the hospital information system and medical records system. The following data of the included patients were recorded: sex, age, height, weight, body mass index, history of splenectomy, hematologic tumors, hypertension, hyperlipidemia, long-term drug use (such as aspirin, nifedipine, hydroxyurea, etc.) and coagulation function, including the prothrombin time (PT), international normalized ratio (INR), partial thromboplastin time ratio (PTR), prothrombin activity (PTA), activated partial thromboplastin time (APTT), fibrinogen (FIB), thrombin time (TT) and D-dimer. Coagulation parameters were only available for inpatients, and only pre-treatment coagulation parameters were collected to reflect the association between extreme thrombocytosis and coagulation function.

### Etiological analysis of patients with extreme thrombocythemia

The etiology of each patient with extreme thrombocytosis was independently determined by two hematology physicians according to the patient's medical information. The physicians would review the medical history and laboratory results, and the principle etiology would be identified by evaluating the characteristic manifestation of specific disease, the time-event relationship, treatment response and all available information. Any discrepancies were resolved by discussion.

### Statistical analysis

The demographic characteristics of the included patients were descriptively summarized. The continuous data are presented as the means ± standard deviations or medians with quartiles, and the categorical data are presented as numbers and percentages. We compared the differences in etiology among patient subgroups stratified by age, sex and department. The proportions of inpatients with low, normal, high and critical values of each coagulation indicator were calculated to explore the influence of extreme thrombocytosis on the coagulation function of patients. Only inpatients' coagulation functions were analyzed, as these indicators were only available for these patients. All the statistical analyses were performed via SPSS Statistics 25. The graphical visualizations were performed with Origin2021.

## Results

### Patient inclusion and characteristics

Finally, 437 patients, consisting of 254 inpatients, 125 outpatients, and 58 emergency patients, were included in the study. The demographic characteristics of the included patients are shown in Table 1.

**Table 1:** Demographic characteristics of the included patients.

**Variable**	**Total**	**Inpatients**	**Outpatient**	**Emergency**	***P***
**Sex**, ***n*** **(%)**					
Male	233 (53.3%)	147 (57.9%)	60 (48%)	26 (44.8%)	0.072
Female	204 (46.7%)	107 (42.1%)	65 (52%)	32 (55.2%)	0.064
Age (years)	50.2 ± 29.4	45.6 ± 30.8	51.3 ± 25.0	66.0 ± 24.6	0.057
Height (cm)	143.7 ± 37.5	134.5 ± 43.9	156.7 ± 19.5	157.7 ± 17.2	0.966
Weight (kg)	45.5 ± 23.9	42.0 ± 24.6	55.0 ± 21.2	54.3 ± 17.4	0.001
BMI (kg/m^2^)	21.1 ± 4.6	20.6 ± 4.1	22.0 ± 5.4	21.5 ± 3.2	0.063
PLT (10^9^/L)	1,223.8 ± 300.6	1,195.25 ± 247.05	1,282.54 ± 408.74	1,222.33 ± 213.26	0.029
History of splenectomy	48 (11.0%)	37 (14.6%)	9 (7.2%)	2 (3.4%)	0.003
Hematologic tumor	58 (13.3%)	23 (9.1%)	28 (22.4%)	7 (12.1%)	0.002
Hypertension	145 (33.2%)	80 (31.5%)	34 (27.2)	31 (53.4%)	0.022
Diabetes	16 (3.7%)	12 (4.7%)	2 (1.6%)	2 (3.4%)	0.464
Hyperlipidemia	3 (0.7%)	3 (1.2%)	0	0	–
Aspirin	41 (9.4%)	21 (8.35%)	10 (8%)	10 (17.2%)	0.385
Nifedipine	30 (6.7%)	15 (5.9%)	7 (5.6%)	8 (13.8%)	0.089
Hydroxyurea	18 (4.1%)	8 (3.1%)	4 (3.2%)	6 (10.3%)	0.007

BMI, body mass index; PLT, platelet count.

### Etiology of overall patients

The etiology of extreme thrombocytosis was analyzed by physicians, and the distribution of etiology in all patients with extreme thrombocytosis is shown in Table 2. The most common cause was myeloproliferative neoplasm (MPN), followed by infection and asplenia. Notably, the etiology for nearly 10% (41/437) of patients is unclear.

**Table 2:** The etiology of extreme thrombocytosis of included patients.

**Etiology**	**Number**	**Percent/%**
Myeloproliferative neoplasm	216	49.4
Infection	56	12.8
Asplenia	52	11.9
Chronic myelogenous leukemia	43	9.84
Medications	8	1.83
Others	21	4.81
Unknown	41	9.38

### Comparison of etiologies among various subgroups

**Figure 1:**
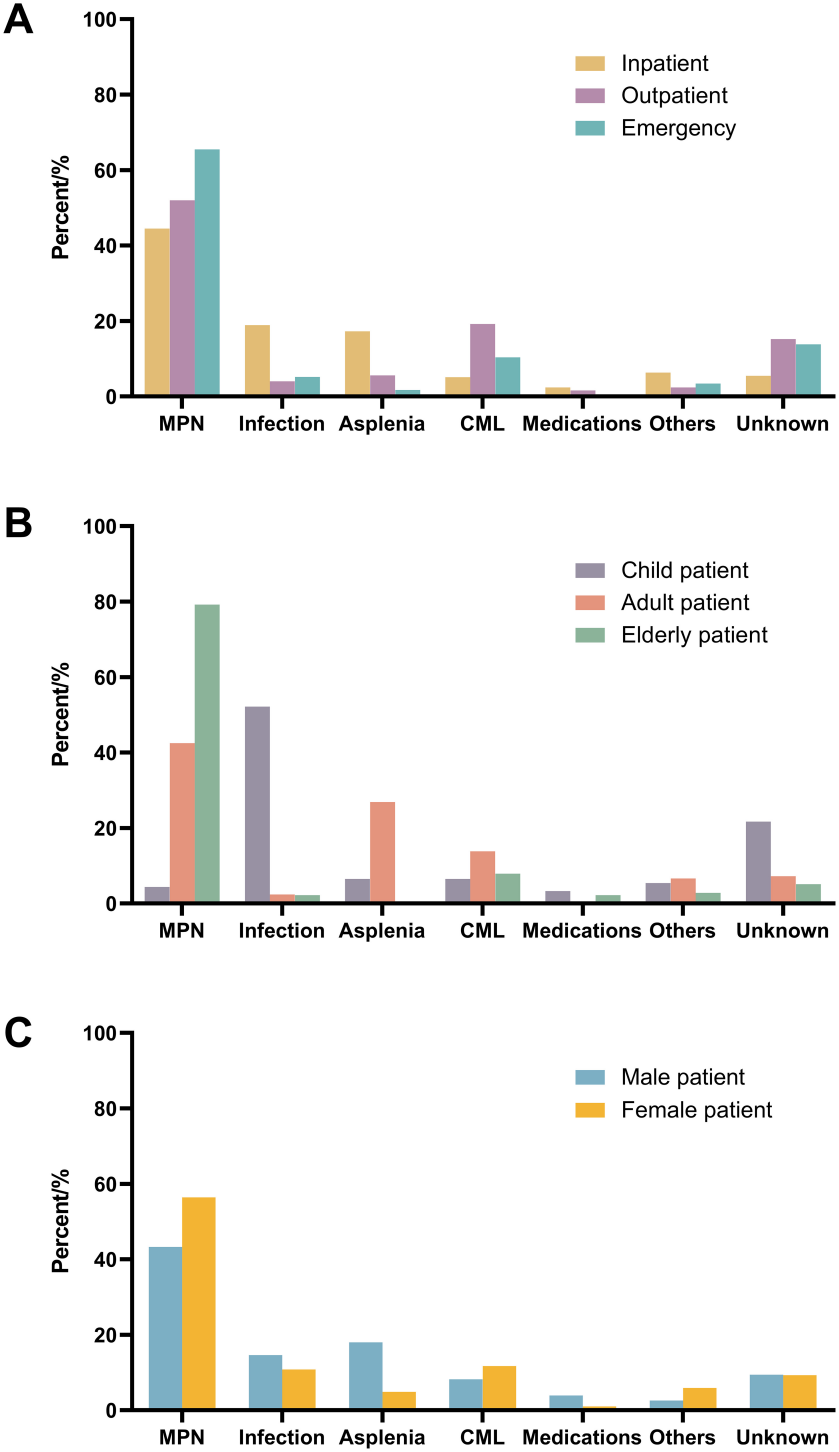
Comparison of etiology of different patient subgroups. **(A)** Patients from different department; **(B)** patients at different age; **(C)** patients with different sex. MPN, myeloproliferative neoplasm; CML, chronic myelogenous leukemia.

We compared the etiologies of patients from different departments and reported that the most common etiology in all subgroups was MPN, and the rarest etiology was medications (Figure 1A). The second most common etiologies for inpatients, outpatients and emergency patients were infection, chronic myelogenous leukemia (CML), and unknown, respectively.

We also compared the etiologies of patients at different ages (Figure 1B). Although the main etiology of both adult and elderly patients was MPN, elderly patients accounted for much higher percentages (nearly 80%). Infection is the main etiology of extreme thrombocytosis in children.

The etiologies of females and males are similar, except for asplenia (Figure 1C). The percentage of asplenia is much greater in males.

### Coagulation function in hospitalized patients with extreme thrombocytosis

In this study, the pre-treatment coagulation function of hospitalized patients with extreme thrombocytosis only was analyzed, and the results are shown in Table 3. The reference ranges of various coagulation function indices are shown in Supplementary Table S1. PT abnormalities were more common in patients with extreme thrombocytosis. A total of 87.5% of the patients had PT that were greater than the normal range. D-dimer and TT abnormalities were followed PT abnormalities at percentages of 60.1 and 56.9%, respectively. The INR and PTR were not commonly affected by extreme thrombocytosis, and 95.4 and 78.4% of the patients had INR and PTR values within the normal range, respectively. Notably, APTT and FIB were in critical condition for 11.1 and 13.7% of the patients, respectively.

**Table 3:** Index of coagulation function in hospitalized patients with extreme thrombocytosis.

**Coagulation function**	**Lower**	**Normal**	**Higher**	**Critical value**
PT	0	19 (12.5%)	133 (87.5%)	
INR	0	145 (95.4%)	7 (4.6%)	
PTR	0	120 (78.4%)	33 (21.6%)	
PTA	31 (20.3%)	102 (66.7%)	20 (13.0%)	
APTT	2 (1.3%)	75 (49.0%)	59 (38.6%)	17 (11.1%)
FIB	4 (2.6%)	76 (49.7%)	52 (34%)	21 (13.7%)
TT	0	66 (43.1%)	87 (56.9%)	
D-dimer	0	61 (39.9%)	92 (60.1%)	

Coagulation function parameters were collected in pre-treatment inpatients. The patient number of each parameter may vary due to the data availability.

PT, prothrombin time; INR, international normalized ratio; PTR, partial thromboplastin time ratio; PTA, prothrombin activity; APTT, activated partial thromboplastin time; FIB, fibrinogen; TT, thrombin time.

## Discussion

To the best of our knowledge, this was the first single-center retrospective study to analyze the etiology of extreme thrombocytosis in different populations and the effects of extreme thrombocytosis on coagulation function of inpatients. Our study provides a new perspective on the etiology of extreme thrombocytosis and could be helpful for the future management of extreme thrombocytosis.

The etiology of extreme thrombocytosis in all populations has been analyzed in some studies. Surgical complications are the main cause of, followed by hematologic malignancies without recent surgery (14, 15). Infection, inflammation, spleen insufficiency, drug and iron deficiency are rare causes (10, 16, 17). However, there are also different views. Another study that analyzed the etiology of thrombocytosis in 801 patients revealed that infection is a common cause of thrombocytosis (18). In a single-center retrospective study of 1,202 patients, secondary thrombocytosis was substantially more common than primary thrombocytosis, and the most common cause of secondary thrombocytosis was tissue damage (10). Previous studies have ignored differences in causes between different groups, leading to different conclusions. A single-center study described the etiology and clinical course in children with severe thrombocytosis (PLT > 900 × 10^9^/L) and extreme thrombocytosis (PLT > 1,000 × 10^9^/L) in a tertiary pediatric hospital (9). This study revealed a low proportion of primary thrombocytosis and a low incidence of thrombosis in children with severe thrombocytosis and extreme thrombocytosis and suggested that these patients may share common characteristics but may have features that are distinct from those in adults. Our study performed a comparative analysis of the etiology among different groups, making the results more accurate and intuitive. Although we had tried our best to determine the cause, the etiologies of some extreme thrombocytosis patients were unknown. Maybe parts of these patients were primary thrombocytosis. But it also reflects the shortage in management of these patients. The more expensive techniques, such as molecular workup and marrow examination were seldomly used for the cause of thrombocytosis. This study also calls more efforts in identify the etiology of extreme thrombocytosis in future.

In addition, our study analyzed the effects of extreme thrombocytosis on coagulation function. The main concern of extreme thrombocytosis is its impact on coagulation function, as bleeding or thromboembolism events can be fatal (12, 19). However, there are few data available at present. A previous study revealed that tissue factor levels are elevated in patients with essential thrombocytosis and that the TF-dependent blood coagulation pathway is activated (20). However, coagulation function has not been reported. Polokhov evaluated platelet function and blood coagulation system status in childhood essential thrombocythemia and reported that the activated partial thromboplastin time was slightly prolonged, the prothrombin index was slightly shortened and thrombin time was normal, whereas fibrinogen was mildly decreased in these patients (21). Zulkafli et al. reported a 5-year-old boy with extreme thrombocytosis, but his coagulation function was within the normal range (22). This study analyzed the coagulation function of extreme thrombocytosis patients and revealed that extreme thrombocytosis had a greater impact on PT and a lesser impact on the INR and PTR, but some patients had APTT and FIB in critical condition. The pathological explanation for PT prolongation under extreme thrombocytosis is unclear, but some study attributed the reason to abnormal platelet function (21). APTT and FIB are important biomarkers for bleeding or thromboembolism events, and the abnormalities should be taken concern. This result could benefit the future management of extreme thrombocytosis.

This study has several limitations. Some bias may exist because of the retrospective, observational nature of the current study. This study is a single-center study that only includes patients in one hospital, and it should be take caution when generalizing the findings. Coagulation parameters were only available for part of inpatients. Some important laboratory test results, such as ferritin levels, were not feasible. Finally, the management and outcome of extreme thrombocytosis are not known but should be assessed in future studies.

## Conclusion

This was a single-center retrospective study to investigate the etiology of patients with extreme thrombocytosis, as well as subgroups. This study revealed that the most common cause of hospitalization and outpatient emergency patients was MPN. The most common cause in children is infection, and the most common cause in adults is asplenia. In terms of coagulation function for inpatients, PT abnormalities were common in patients with extreme thrombocytosis, but INR abnormalities were unusual. However, APTT and FIB were under critical condition for some patients. This study provides an important basis for the diagnosis and treatment of extreme disease and emphasizes the importance of changes in the coagulation function of these patients.

## Data Availability

The original contributions presented in the study are included in the article/Supplementary material, further inquiries can be directed to the corresponding author.

## References

[1] Liaskas A Vassilakopoulos TP. Thrombocytosis. Blood. (2024) 143:1782. 10.1182/blood.2023023702 38662384

[2] Harrison CN Bareford D Butt N Campbell P Conneally E Drummond M. Guideline for investigation and management of adults and children presenting with a thrombocytosis. Br J Haematol. (2010) 149:352–75. 10.1111/j.1365-2141.2010.08122.x 20331456

[3] Stockklausner C Duffert CM Cario H Knöfler R Streif W Kulozik AE. Thrombocytosis in children and adolescents-classification, diagnostic approach, and clinical management. Ann Hematol. (2021) 100:1647–65. 10.1007/s00277-021-04485-0 33712866 PMC8195939

[4] Godfrey AL Green AC Harrison CN. Essential thrombocythemia: challenges in clinical practice and future prospects. Blood. (2023) 141:1943–53. 10.1182/blood.2022017625 36379024

[5] Kucine N Chastain KM Mahler MB Bussel JB. Primary thrombocytosis in children. Haematologica. (2014) 99:620–8. 10.3324/haematol.2013.092684 24688110 PMC3971071

[6] Sulai NH Tefferi A. Why does my patient have thrombocytosis? Hematol Oncol Clin North Am. (2012) 26:285–301, viii. 10.1016/j.hoc.2012.01.003 22463828

[7] Venkat RK Redd RA Harris AC Aryee MJ Marneth AE Kamaz B. Risk of bleeding in patients with essential thrombocythemia and extreme thrombocytosis. Blood Adv. (2024) 8:6043–54. 10.1182/bloodadvances.2024013777 39293089 PMC11635702

[8] Hsieh RW Ravindran A Hook CC Begna KH Ashrani AA Pruthi RK. Etiologies of extreme thrombocytosis: a contemporary series. Mayo Clin Proc. (2019) 94:1542–50. 10.1016/j.mayocp.2019.01.041 31378229

[9] Kishimoto K Hasegawa D Nakagishi Y Kurosawa H Tanaka T Hatakeyama T. Etiology and clinical course of severe and extreme thrombocytosis in children: a retrospective single-center study. Eur J Pediatr. (2024) 183:4783–8. 10.1007/s00431-024-05755-5 39227506

[10] Edahiro Y Kurokawa Y Morishita S Yamamoto T Araki M Komatsu N. Causes of Thrombocytosis: a single-center retrospective study of 1,202 patients. Intern Med. (2022) 61:9282–21. 10.2169/internalmedicine.9282-21 36385045 PMC9751737

[11] Galvez C Stein BL. Thrombocytosis and thrombosis: is there really a correlation? Curr Hematol Malig Rep. (2020) 15:261–7. 10.1007/s11899-020-00588-z 32399765

[12] Kvernberg J Grove EL Ommen HB Hvas A-M. Platelet function and turnover in essential thrombocythemia: a systematic review. Semin Thromb Hemost. (2021) 47:90–101. 10.1055/s-0040-1718873 33525042

[13] Marxsen JH Forchheim S Zuske-Matthäus A Wagner T. Prevalence of platelet dysfunction and abnormal coagulation: results of a population-based study. Clin Appl Thromb. (2009) 15:421–7. 10.1177/1076029608315164 18387983

[14] Tefferi A Vannucchi AM Barbui T. Essential thrombocythemia: 2024 update on diagnosis, risk stratification, and management. Am J Hematol. (2024) 99:697–718. 10.1002/ajh.27216 38269572

[15] Gurrieri C Smith BB Nuttall GA Pruthi RK Said SM Smith MM. Essential thrombocythemia and cardiac surgery: a case series and review of the literature. Ann Thorac Surg. (2018) 106:482–90. 10.1016/j.athoracsur.2018.03.057 29705369

[16] Chen H Xu X Li P Xu Z. Imipenem–cilastatin-induced thrombocytosis: a probable rare case report. Eur J Inflamm. (2022) 20:1–4. 10.1177/1721727X221078719

[17] Vo QT Thompson DF. A review and assessment of drug-induced thrombocytosis. Ann Pharmacother. (2019) 53:523–36. 10.1177/1060028018819450 30525921

[18] Rose SR Petersen NJ Gardner TJ Hamill RJ Trautner BW. Etiology of thrombocytosis in a general medicine population: analysis of 801 cases with emphasis on infectious causes. J Clin Med Res. (2012) 4:415–23. 10.4021/jocmr1125w 23226175 PMC3513424

[19] Jimenez K Leitner F Leitner A Scharbert G Schwabl P Kramer A-M. Iron deficiency-induced thrombocytosis increases thrombotic tendency in rats. Haematologica. (2021) 106:782–94. 10.3324/haematol.2019.245092 32079699 PMC7928018

[20] Gadomska G Ziołkowska K Boinska J Filipiak J Rość D. Activation of TF-dependent blood coagulation pathway and VEGF-A in patients with essential thrombocythemia. Medicina. (2019) 55:54. 10.3390/medicina55020054 30781507 PMC6409549

[21] Polokhov DM Ershov NM Ignatova AA Ponomarenko EA Gaskova MV Zharkov PA. Platelet function and blood coagulation system status in childhood essential thrombocythemia. Platelets. (2020) 31:1001–11. 10.1080/09537104.2019.1704710 31856623

[22] Zulkafli Z Janaveloo T Wan Ab Rahman WS Hassan MN Abdullah WZ. Extreme thrombocytosis in a child: laboratory approaches and diagnostic challenges. Oman Med J. (2019) 34:336–40. 10.5001/omj.2019.65 31360323 PMC6642721

