# Supplementary material for: Etiology of patients with extreme thrombocytosis and its association with coagulation function: 10-year experience in a tertiary hospital
Source: Front Med (Lausanne). 2025 Oct 23;12:1650704. doi: 10.3389/fmed.2025.1650704 (PMC12588944; doi:10.3389/fmed.2025.1650704)
Supplement: Supplementary file 1 [file Data_Sheet_1.DOCX]

|  | Low | Normal | High | Critical value |
| --- | --- | --- | --- | --- |
| PT (s) | ＜11 | 11-13 | ＞13 | ＞30 |
| INR | ＜0.8 | 0.8-1.5 | ＞1.5 | ≥4 |
| PTR (s) | ＜0.82 | 0.82-1.15 | ＞1.15 |  |
| PTA (%) | ＜75 | 75-100 | ＞100 |  |
| APTT (s) | ＜25 | 25-37 | ＞37 | ＜20 or ＞80 |
| FIB (g/L) | ＜2 | 2-4 | ＞4 | ＜1 |
| TT (s) | ＜12 | 12-16 | ＞16 | ＞100 |
| D-dimer (mg/L) |  | 0-0.5 | ＞0.5 | ＞1.5 |

Table S1 The reference range of coagulation function indicators

PT, prothrombin time; INR, international normalized ratio; PTR, partial thromboplastin time ratio; PTA, prothrombin activity; APTT, activated partial thromboplastin time; FIB, fibrinogen; TT, thrombin time.
